# Stress Evaluation in Simulated Autonomous and Manual Driving through the Analysis of Skin Potential Response and Electrocardiogram Signals

**DOI:** 10.3390/s20092494

**Published:** 2020-04-28

**Authors:** Pamela Zontone, Antonio Affanni, Riccardo Bernardini, Leonida Del Linz, Alessandro Piras, Roberto Rinaldo

**Affiliations:** Polytechnic Department of Engineering and Architecture, University of Udine, Via delle Scienze 206, 33100 Udine, Italy; pamela.zontone@uniud.it (P.Z.); antonio.affanni@uniud.it (A.A.); riccardo.bernardini@uniud.it (R.B.); dellinz.leonida@spes.uniud.it (L.D.L.); piras.alessandro@spes.uniud.it (A.P.)

**Keywords:** autonomous driving, stress recognition, skin potential response, electrocardiogram, supervised learning algorithm

## Abstract

The evaluation of car drivers’ stress condition is gaining interest as research on Autonomous Driving Systems (ADS) progresses. The analysis of the stress response can be used to assess the acceptability of ADS and to compare the driving styles of different autonomous drive algorithms. In this contribution, we present a system based on the analysis of the Electrodermal Activity Skin Potential Response (SPR) signal, aimed to reveal the driver’s stress induced by different driving situations. We reduce motion artifacts by processing two SPR signals, recorded from the hands of the subjects, and outputting a single clean SPR signal. Statistical features of signal blocks are sent to a Supervised Learning Algorithm, which classifies between stress and normal driving (non-stress) conditions. We present the results obtained from an experiment using a professional driving simulator, where a group of people is asked to undergo manual and autonomous driving on a highway, facing some unexpected events meant to generate stress. The results of our experiment show that the subjects generally appear more stressed during manual driving, indicating that the autonomous drive can possibly be well received by the public. During autonomous driving, however, significant peaks of the SPR signal are evident during unexpected events. By examining the electrocardiogram signal, the average heart rate is generally higher in the manual case compared to the autonomous case. This further supports our previous findings, even if it may be due, in part, to the physical activity involved in manual driving.

## 1. Introduction

Increasingly sophisticated assisted and autonomous vehicles are becoming readily available. Autonomous driving is categorized in relation to the human involvement during driving. It varies from level zero, where the driver controls everything in the car and there is no automation, up to level five, where there is full automation of the control, and the expectation is that the performance of the autonomous system matches the one of a human driver. The levels of automation two and three have been considered as reached goals just a couple of years ago, and the route to fully autonomous cars seemed within easy reach. In reality, the impact of assisted or autonomous cars on humans (either drivers or passengers) is not predictable. In this scenario, there is an increasing interest in the technologies that provide real-time monitoring of the driver psycho-physiological reactions to vehicle dynamics under direct or automated control. Among the aspects that can be examined, the study of the human’s robotic acceptance, the evaluation of the difference between self and assisted/autonomous driving in the same scenarios, and the analysis of the impact of on-the-fly setup variations of the vehicle, when it is autonomously driven, are of paramount interest.

The psychological and mental state of both drivers and passengers is critical in autonomous driving as well as in manual driving. As far as manual driving is concerned, stress and arousal monitoring in car drivers is being largely studied [1,2,3,4]. In literature, we can find systems that use physical measurements [5,6], physiological measurements [7,8,9,10], or both, along with data regarding the environment [11,12], the vehicle [13], and the driver’s movements [14]. In the aforementioned works, several implementations of classifiers are presented, especially the ones based on Machine Learning (ML), which is the most popular approach [15]. ML is used to recognize human expressions and behaviors [5,16], and physiological characteristics [14,17,18,19].

Considering autonomous driving, in [20], authors have provided a chauffeur to the subjects, who plays the role of an autonomous driving system, in order to monitor their behavior during the car trips when they do not drive. With a chauffeur, they are more willing to sustain a greater number of trips and a longer traveled distance, compared to the case when they have to manually drive. This happens when a person is not worried about the driver’s behavior, but autonomous driving systems have not reached such a perceived reliability yet. So, the analysis of the passenger’s psychological state is needed to evaluate the level of trust that people place in autonomous driving systems, and the social impact of this new technology.

Switching to manual driving can always occur, for example, when a pedestrian suddenly crosses the street, especially in dense traffic [21]. Authors in [22] show how the vehicle’s sensors performance deteriorates during adverse weather conditions. Other studies have shown how peoples drowsiness can rise during autonomous driving, deteriorating their reactions when they have to take control of the vehicle [23]. Even if the drivers have to monitor the driving system, after some time, their attention decreases, and in some cases, they even fall asleep [24]. This brought the researchers to search for tasks that do not make people get drowsy during trips, and that at the same time do not distract them, so they are still able to act when it is necessary [25,26]. Authors in [27] consider eye-tracking techniques for highlighting pedestrians and other critical objects when the subjects are distracted by the tasks assigned to them during the trip. In [28], authors present an advanced eye-tracking system, which is able to analyze and classify, according to their importance, multiple events at the same time in an automated driving scenario. In [29,30], electroencephalography (EEG) is employed to check the drowsiness and the emotional state of passengers in self driving vehicles.

Our main contribution in this work is the design of a specific methodology to evaluate the reactions and emotional states of subjects who drive in a simulated environment, during manual and autonomous driving. This paper extends the work presented in [31] by considering the use of ML techniques, which were not considered there. We analyze the two different scenarios, autonomous and manual driving, with some stress inducing episodes happening along a track. The tests are conducted in a company [32] using its own designed professional driving simulator (DiM 150). In particular, the driving simulator uses several different Advanced Driver Assistance Systems (ADAS) algorithms. In autonomous driving, the Automatic Emergency Braking (AEB) activates the brakes in case the car is approaching another vehicle too fast. The Adaptive Cruise Control (ACC) monitors the distance to the vehicle in front, and if this distance decreases under a certain threshold, the cruise speed is reduced. The Lane Keeping Assist (LKA) keeps the vehicle within the lane markings. The Blind Spot Detection (BSD) uses sensors to detect the presence of another vehicle in nearby lanes in case of overtaking. The AutoPilot, on the other hand, autonomously controls the vehicle dynamics, such as the steering wheel, accelerator, and brake, for a completely autonomous drive. All of these controls are disabled during manual driving.

We show that the subjects’ SPR signal is more often classified as “stress” during the whole drive in the manual driving scenario, compared to the autonomous driving scenario. In the autonomous drive, we notice that few time intervals are classified as “stress” during the drive. However, the intensity of SPR peaks when performing the tasks is higher in the autonomous scenario compared to the manual driving scenario. During the experiment, we also acquire the electrocardiogram (ECG) signal of the subjects. We use it to calculate the mean heart rate of the subjects, finding that the heart rate is higher during the manual driving scenario. This suggests that the stress level is also higher, even if the heart rate increase may be due in part to the physical activity involved in manual driving (see [33] for example). These preliminary results can give useful insights in vehicle development about autonomous and assisted driving, and about the efficiency and comfort level of driving assistance devices in realistic highway driving scenarios.

## 2. Methods

**Figure 1:**
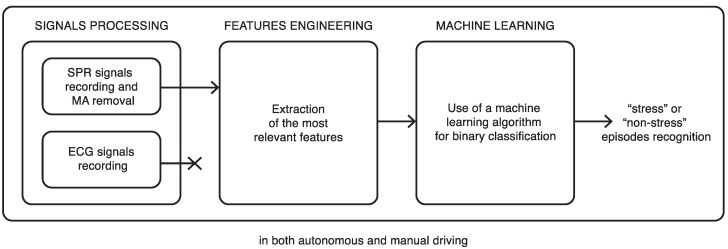
The proposed scheme: the processing blocks are the same in both the manual and autonomous driving scenarios.

In this section, we introduce the fundamental blocks of our system, as presented in Figure 1. The first block includes the recording process of the two Skin Potential Responses (SPRs) from each hand of the tested subject, and of the electrocardiogram signal registered from the chest. The sensor utilized to record these physiological signals will also be briefly introduced in this section. The two SPRs are then sent as input to the Motion Artifact (MA) Removal algorithm that has already been presented in [34,35]. In fact, it is well known that motion artifacts due to vibrations or movements of the hands on the steering wheel appear during the drive and can alter the signal morphology [36,37]. In the autonomous scenario, the subjects were told to rest their hands on the knees, and we dot not expect any MA in the SPR signals. Motion artifacts were not present in the ECG signal, due to the use of a commercial vest with wet electrodes and adhesive conductive gel, which prevented possible signal alterations due to motion. In the experiment, that will be described in Section 3, we generate some stress episodes in the individuals by subjecting them to a sequence of stress inducing events (the same for every subject), in different locations and timing along a simulated highway drive, in both autonomous and manual scenarios. Some specific features are extracted from the SPR signal resulting from the MA removal step. These features are used as input to an ML classification algorithm that allows the recognition of stress and normal driving episodes in all of the subsequent time intervals analyzed in the experiment. We are thus able to identify and compare the emotional reactions generated by the tasks, which may induce different stress responses in autonomous compared to manual driving.

### 2.1. Signals Processing

**Figure 2:**
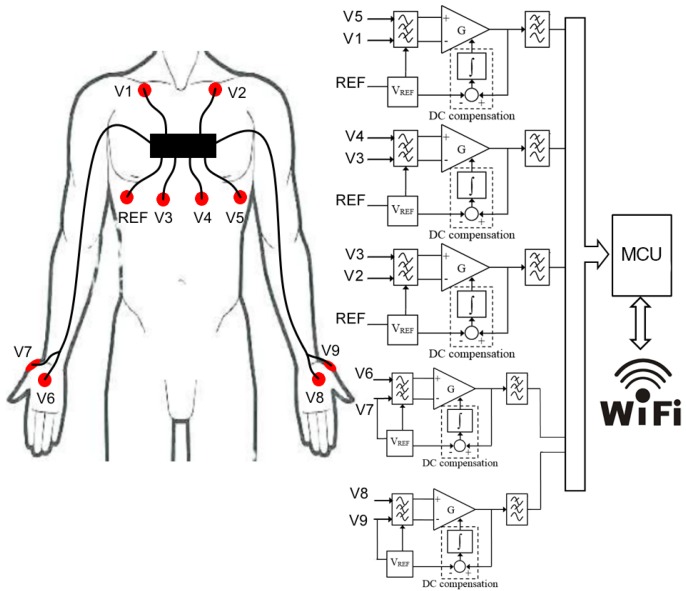
Electrodes arrangement and sensor block diagram.

Here we present the sensor used to record both the ECG and SPR signals, and we briefly describe the MA removal algorithm. The sensor is the main component of the VI-BioTelemetry designed by the VI-grade company. The block diagram is shown in Figure 2. The SPR signals are measured between the palm (V6 and V8 in Figure 2) and the back (V7 and V9 in Figure 2) of the hands using Ag/AgCl disposable electrodes. Three ECG channels are acquired using a commercial vest with wet textile electrodes positioned as shown in Figure 2. A voltage reference VREF is applied on the chest and on V7 and V9.

The differential voltages V5-V1, V4-V3, V3-V2, V6-V7, V8-V9 (3 ECG and 2 SPR channels) are amplified and conditioned by the analog section of the sensor. A low-power microcontroller unit (STM32 MCU) converts the conditioned analog signals into digital data (using the on-board A/D converter) and transmits them to a laptop by means of a WiFi module. The sensor is supplied using a single 3.7 V, 1000 mAh, Li-Ion cell lithium polymer battery. Since the sensor current consumption is 200 mA during transmission, it is possible to acquire data for 5 h of continuous operation. The power supply section of the sensor converts the battery cell voltage down to +3.3 V by means of a buck DC-DC converter. The reference voltage VREF = 1.65 V applied to the reference electrodes is provided by a linear voltage reference. Details about both the SPR and the ECG circuit designs are reported in [38,39]. The conditioned analog signals are then sent to an analog input of the MCU, which has on board a 12 bit A/D converter that acquires the data at 1 kSa/s sample rate. The converted data are sent via the SPI protocol to the WiFi module. The system is designed for 3 channels acquisition, as shown in Figure 2. We chose a multi channel system for greater flexibility and because we are able to select the derivation with a higher signal to noise ratio (which is, typically but not always, the V3-V2 derivation). Moreover, the electrodes placement and arrangement is standard, dictated by the characteristics of the commercial vest.

**Figure 3:**
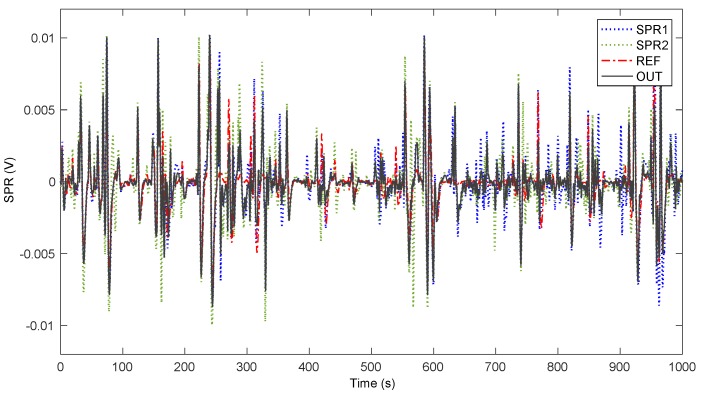
Comparison of the signals acquired from the two hands, the reference signal and the output of the proposed algorithm in the Motion Artifact (MA) test.

**Figure 4:**
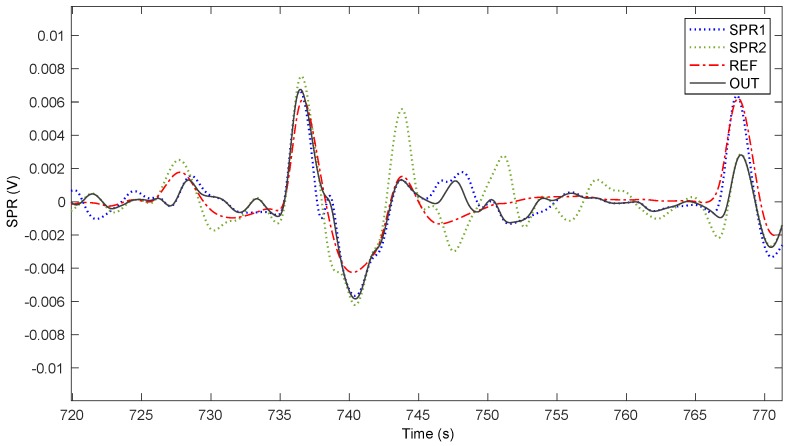
Detail of a section of Figure 3.

In [34], we described a MA removal algorithm used to remove the artifacts that the driver’s hands movements introduce during a drive, which could alter the measured SPR signals. It takes as inputs the two different SPR signals coming from both driver’s hands, and it outputs a single cleaned SPR signal. It works on the hypothesis that the perturbations due to motion produce an increase of the energy in the recorded signals, because there will be a contribution provided by actual Electrodermal Activity (EDA) measurements, and another contribution due to the hand movement. So, the output signal is obtained as a weighted combination of the two signals, based on their local energy, and follows the “smoother” SPR signal, which is the input signal characterized by the lowest level of energy. We assume that motion artifact is present in only one of the two signals at a time, even when the driver holds the steering wheel with both hands. This is due to the fact that MA is determined, at least in the majority of situations, when the steering wheel is turned left or right, thus engaging one of the two hands at a time. This supposition is frequently confirmed by analyzing actual data, there are instances where this does not happen, but still the proposed algorithm appears to be a reasonable solution to reduce MA. To evaluate the extent of the applicability of our proposed solution we designed a test (see also [35]), adding two novel sensors, applied to each hand, which record at the same time the SPR on the outer region of the palms, which is a zone less perturbed by motion artifacts. This new sensors setup was designed for the test, even if it requires special driving care, in order to not displace the sensors, and cannot be used during normal driving. The two supplementary sensors were posed on the outer part of the hand (roughly, over the *abductor digiti minimi* muscle) and the driver was grasping the steering wheel only with the inner part of the hand using thumb and forefinger (roughly, using the *abductor policis* muscle). In this unnatural way of driving, we obtained four signals: two of them (the ones close to thumb) were affected by motion artifact and the other two (the ones close to the little finger) never touched the steering wheel remaining still at their original position. Thus, these latter signals were not perturbed by any motion of the hand. From the average of these signals, we extracted the reference signal (REF), which should not evidence MA. The full test lasted 18 min and encompassed one subject driving freely on a road with straights and turns, where the SPR from the novel sensors was recorded along with the old ones. Figure 3 shows the signals acquired from the two hands (SPR1 and SPR2), together with the reference signal (REF), and the output of the proposed algorithm (OUT). In the case of a motion artifact appearing in one hand only, the system generates an output signal that is similar to the one resulting from the mean of the signals recorded from the novel sensors less prone to motion artifacts, thus working as planned. The instances where motion artifacts appear at the same time in both hands are limited (roughly 4 occurrences in the 18 min long experiment, for a total duration of about 20 s), and when this happens, the system might generate a false positive stress recognition. However, the frequency of these situations, as noticed, is generally low. Figure 4 shows a detail of the signals in the time interval 720–770 s. It is apparent that the OUT signal follows the REF signal more closely, despite the possibility that MA occurs in one of the two hands or simultaneously with an opposite sign. From a quantitative point of view, the RMS values of the traces in Figure 3 are REF = 1.8 mV, SPR1 = 2.4 mV, SPR2 = 2.5 mV, OUT = 1.9 mV. Thus, the possible artifact contribution to OUT is in the order of 5%. We analyze the difference between the REF and OUT signals during the whole experiment, noticing that the variance of the resulting signal is 1.51 mV^2^, that is smaller than the variance obtained calculating the difference between REF and SPR1 (2.08 mV^2^), and considerably smaller than the variance obtained calculating the difference between REF and SPR2 (2.92 mV^2^).

### 2.2. Features Engineering and Machine Learning

As introduced before, we use the data collected from the subjects during the driving in the autonomous and manual scenarios, to test a classification ML algorithm and collect the performance results. In particular, we use a Support Vector Machine (SVM) model trained on data coming from a manual drive experiment, conducted in the same company and using the same professional simulator. In other previous experiments (see [40] as an example), we compared different ML algorithms, and we found out that the performance was similar, so in this work, we chose the SVM classifier as a convenient solution.

We then utilize the resulting model to test the classifier on the signals recorded in this new setup, where both manual and autonomous driving are considered. Note that our purpose is now to compare each driver’s response in two different situations, manual and autonomous driving, and we aim to do this by using an ML classifier that is able to reliably detect stress events and was trained on a different data set, with more subjects and stress-inducing events.

In detail, the test we performed to build the SVM model involves 18 individuals, 14 male and 4 female, 19–35 years old. The subjects had to drive in a 67 km long track, reproducing a straight highway without traffic, and had to overcome 12 different stress episodes, which were spaced at different locations. The sequence of the 12 stress episodes was randomly generated. Completing the track took about 40 min, as the subjects were instructed to keep a constant velocity between 120 km/h and 130 km/h. Each subject was told to drive normally, as he would do in real life. The 12 events were as follows: Double lane change (right to left or left to right), Tire labyrinth, Sponsor block (from left or from right), Slalom (from left or from right), Lateral Wind (from left or from right), Jersey LR, Tire trap, Stop. The time spent to pass these 12 events was about 15 min, averaging what it took for each subject. Statistical features, which we found to be good indicators of some stress characteristics, were extracted from the processed SPR signal, i.e., the output signal of the MA removal block. In particular, we derived the block variance, the energy, the mean absolute value, the mean absolute derivative, and the maximum absolute derivative. These SPR features, shown in Table 1, were computed considering 15 s long blocks at a time, and were sent as input to the classifier. We analyzed a new block every 5 s, thus overlapping successive intervals. A normalizing procedure was also applied to the SPR signals to make them uniform when different individuals were considered. In particular, we remove the mean value and divide by the signal standard deviation, calculated for the entire track signal.

Being the positions of the obstacles in the track fixed, we knew when and for how long a subject would be stressed. When a block fell outside of these events, we considered it without stress, so “0”, and if a block intersected a stress time interval, we decided to consider it with stress, so “1”. The SVM classifier was set up using the Matlab routine functions (Matlab 2017a) with a Radial Basis Function kernel. The Bayesian optimization procedure has also been applied during the training procedure. We then used the “leave-one-person-out” procedure, so that for the training process we analyzed the data of all subjects, leaving one out, which was the one on which the classifier was tested. A relabeling procedure was also applied to the classifier’s output in order to consider the case of isolated positive stress intervals, as described in [40]. Performance was derived by computing the average of the test results for all of the individuals. We obtained an accuracy of 73%, which confirms the efficiency of the SVM. This 73% accuracy derives from a relatively large number of detected False Positives. Note that, in assessing the classifier’s performance, some assumptions are made that may influence the performance obtained. The first hypothesis assumes that all the signal blocks that are within a time interval in correspondence of an obstacle must highlight stress. In fact, it can happen that a person is not constantly stressed during this interval. The second hypothesis is that the subject is not stressed outside the time intervals corresponding to the obstacles. In general, this may not be true, given that the driver may be subjected, during these intervals, to emotional stimuli that we cannot control. However, since the experiment described in this paper compares manual and autonomous driving in similar contexts, we believe the reported results are consistent.

We only consider the SPR signal features, since we noticed a systematic difference between the characteristics of the instantaneous Heart Rate (HR) between autonomous and manual driving. As we will discuss in Section 4.3, this can be due to the physical activity involved in manual driving, thus making it difficult to compare the two scenarios using HR information.

In order to compare manual and autonomous driving, we normalize the new data, for each subject, considering the standard deviation of the manual driving scenario, as done with the training data (see also [40]). We then extract the same features from the cleaned SPR signal, listed in Table 1, in both manual and autonomous scenarios, which this time are used for testing only. We select each time interval to be 15 s long for testing, picking the next interval after 5 s, thus with an overlap between subsequent intervals. We collect the extracted features to generate the final feature vectors, that values of which are then normalized in the range [0, 1]. In conclusion, we use a previously trained machine learning algorithm to classify all the new extracted intervals in two classes, which are the stress class (or “1”) and the normal driving non-stress class (or “0”). Note that the EDA signals recorded in the normal driving non-stress condition can be rather different from those recorded when a person is at rest or not involved in driving. In the following, we also assume that SPR responses during autonomous and manual driving are directly comparable. The classifier is therefore finally applied to both the autonomous and manual driving SPR signals.

## 3. Experimental Setup

A group composed of 13 healthy volunteers, with an age around 31.4 ± 9 years, 8 males and 5 females, were asked to drive on a professional simulator in a highway road, enduring three different phases. In particular, the test includes an initial warm up phase, common to all of the subjects, a phase with manual driving and one with autonomous driving. In order to avoid data bias, one half of the volunteers did the manual drive before the autonomous one, and the other half vice-versa. The participating subjects gave consent to have physiological signals recorded during the experiments, and the research was carried out following the principles of the Declaration of Helsinki. They were selected among people with different backgrounds. Even if the test pool size is somewhat limited, we think that its heterogeneity is sufficient to keep the bias low.

The initial phase had the aim to instruct people and to get them familiar with the simulator, with very low traffic driving on a highway for 5 min, during which their baseline physiological signals were acquired.

**Figure 5:**
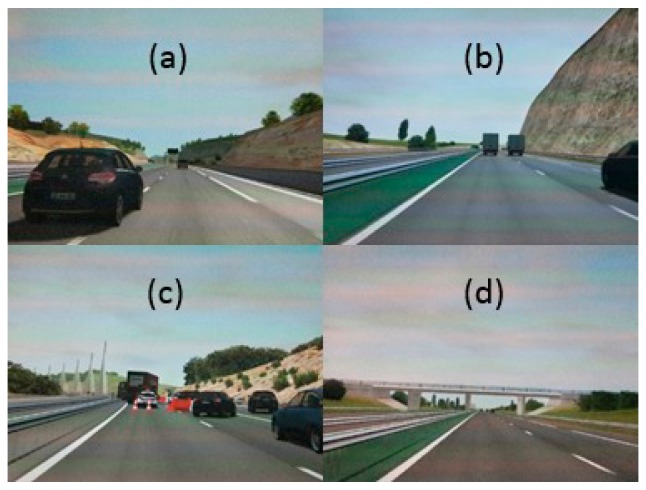
The four tasks encountered by the drivers along the track. (**a**) The “overtake” task. (**b**) The “trucks” task. (**c**) The “narrow” task. (**d**) The “wind” task.

In the manual phase, participants had to manually drive in a highway for 40 km (roughly corresponding to a 20-min drive). On the road, at specified positions, four tasks were to be completed. The first one (“overtake” task) requires to overtake another car, and it is undecided which lane to keep. This task accounts for 3.5 km of the track. The second one (“trucks” task) is a brake maneuver because one truck ahead is overtaking another truck. This task accounts for 2.5 km of the track. The third one (“narrow” task) has a narrowing lane and a mandatory lane shift due to road works. This task accounts for 1 km of the track. In the fourth task (“wind” task), there is an unexpected lateral gust of wind. This task accounts for 5 km of the track. Figure 5 shows screen captures of the tasks.

During the autonomous phase, the subjects were on the same highway but in a simulated autonomous car, which had to complete the same tasks of the manual phase.

**Figure 6:**
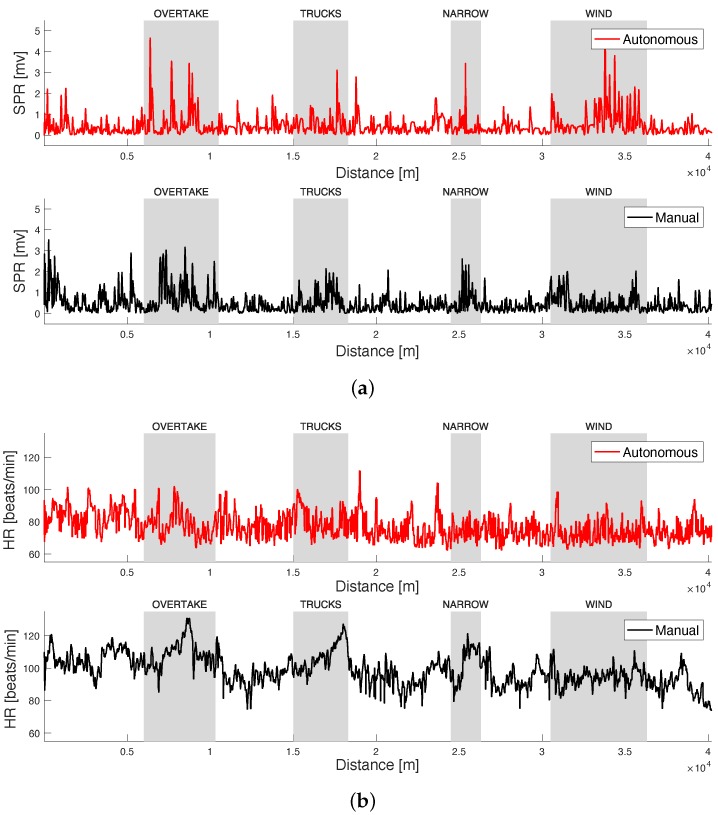
(**a**) The Skin Potential Response (SPR) signal (local RMS value), after the application of the MA removal algorithm, in both driving scenarios. (**b**) The Heart Rate (HR) signal in both driving scenarios. The four areas, corresponding to the stress episodes, are also evidenced.

In Section 4.2, we will report the classification results obtained considering the manual and the autonomous driving phases only. As an example, Figure 6a shows the typical behavior of the SPR signal extracted from a tested subject during the tasks, in the manual and in the autonomous scenarios. In particular, the figure plots the local RMS value of the combined SPR signal. An increase of SPR signal peak amplitude and number is clearly visible in correspondence of the stress inducing tasks. In addition, Figure 6b shows the HR signal recorded from the same subject. The increase of the HR is also evident during the stress inducing tasks, in particular in the manual scenario. As we will see later in Section 4.2, the mean HR of the subjects during all the track in the manual scenario also results to be higher than the mean HR in the autonomous scenario.

## 4. Results

In this section, we discuss the results that we obtain from the tests during the manual and the autonomous driving phases, as introduced in Section 3. Our main objective is to estimate the ability of our system to compare stress levels on the tested subjects, during a simulated manual and autonomous driving session.

### 4.1. Results Considering the SPR Signal Only

**Figure 7:**
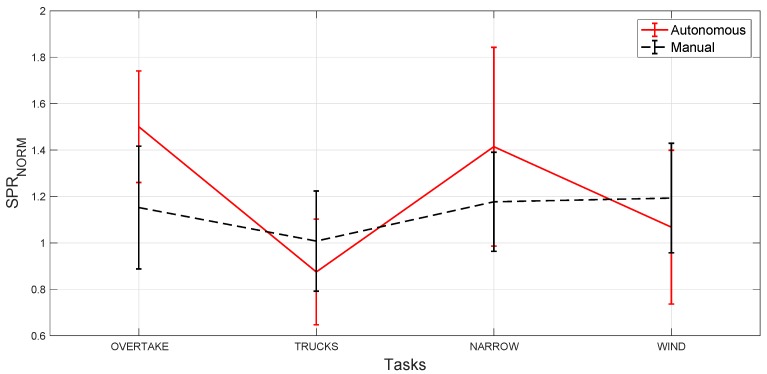
Normalized SPR over the tasks for autonomous (red line) and manual (black line). Error bars represent the standard deviation among the subjects.

As a preliminary analysis, we consider only the SPR signal recorded from the tested subjects, in both scenarios. Our goal is to analyze and find if there are notable differences in terms of SPR activity between each task, among all of the tested subjects. In order to do that, since every subject has a different electrodermal activity signal, we compute the Root Mean Square (RMS) value of the SPR signal for each subject considering only the time intervals corresponding to the different tasks, and then we normalize this value by dividing it by the RMS of the SPR signal computed on the whole driving track. We then calculate the average value of all the normalized SPR results corresponding to all of the subjects. Figure 7 shows the resulting normalized SPR signals for both manual (blackdotted line) and autonomous (red line) driving scenarios. We also use the paired t-test (and the related p-value in %) to evaluate how significant the difference is between the obtained values in the autonomous and manual cases and during the different episodes.

The manual driving scenario results (black dotted line) suggest that the “wind” task is slightly more stressful than the other tasks, and that the “trucks” task is slightly less stressful than all of the other ones, although the normalized SPR value is comparable in all tasks when the subject is driving manually (p > 5% for all the pairs combinations). Instead, during the autonomous driving scenario (red line), the “overtake” task is significantly more stressful compared to the “trucks” task (p = 0.005%) and to the “wind” task (p = 0.6%). Finally, the “trucks” task is significantly less stressful than the “narrow” task (p = 0.6%), which in turn is more stressful than the “wind” task (p = 1%).

Comparing the two scenarios, and performing the t-test for each obstacle, we can see that the “overtake” task is significantly more stressful in autonomous than in manual drive (p = 0.2%). Although non-significant, the “narrow” task is also slightly more stressful in autonomous drive (p = 8%), whereas both the “trucks” and “wind” tasks are stressful in a similar way in the two scenarios (p = 18% and 28%, respectively).

**Figure 8:**
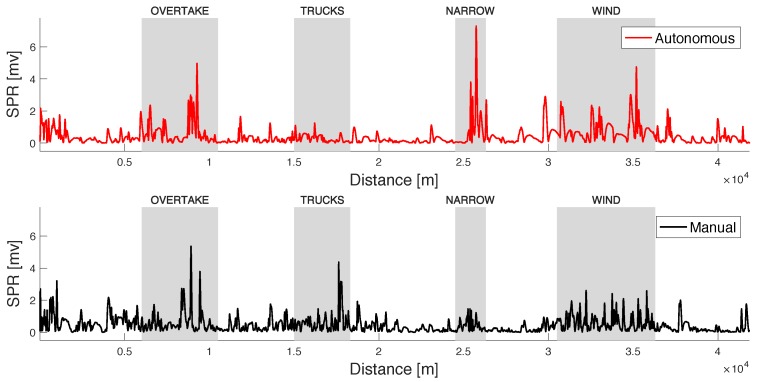
SPR signal of a different test subject, in both manual and autonomous scenarios (local RMS value).

By looking at the plot of the SPR signals of the subjects (see a different example of another subject in Figure 8), we can see that, in the manual scenario, the subjects exhibit during the tasks a larger number of SPR peaks, with a more uniform amplitude, whereas in the autonomous scenario the peaks are less frequent, but may have a higher amplitude when compared to the peaks of the manual scenario. This may suggest that the autonomous drive is less stressful as a whole (and we will prove this later reporting the classifier results), but when the stress is present, it is stronger. Note that, within some inevitable approximation, the discussion above allows to conclude that the increased SPR activity during manual driving, in particular in the regions outside the stress-inducing events, is not due to MA.

### 4.2. SPR Classification Algorithm Results

As already mentioned, we test the data obtained in the experiment comparing manual and autonomous driving by using a machine learning SVM algorithm trained on a larger dataset, containing 3195 examples for each stress and normal driving (non-stress) class.

For the feature extraction process to be consistent with the one used for training, the same features are extracted in our new experiment from 15 s long time blocks of the SPR signal, and then sent to the SVM classification algorithm only for the testing procedure. Every 5 s we consider a new interval, so each interval overlaps the previous one by 10 s.

Each event starts and ends at a specific location on the track. We then know when a subject is supposed to be stressed based on its position on the track. When an interval falls outside of these events, we consider it as “0”, with no stress, and if an interval crosses an event, we count it to be “1”, with stress. Since the individuals can complete the assigned tasks with varying speed and duration, each individual will end up with a different amount of stress/non-stress intervals. In particular, the number of stress intervals we collect for testing can vary among subjects (from 229 to 246).

**Figure 9:**
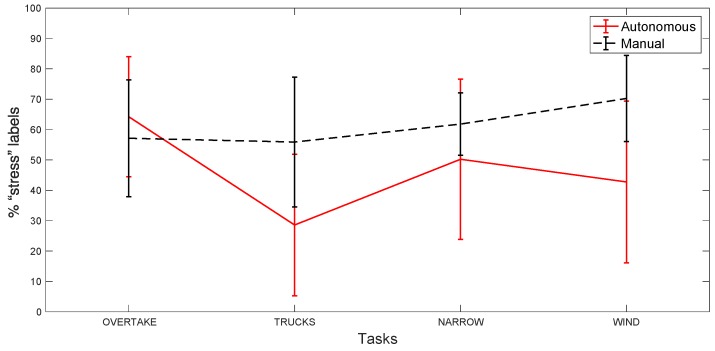
The number of intervals labeled as “stress” of the total of intervals during each task. This is the result of the mean computed considering all of the subjects. Error bars represent the standard deviation among the subjects.

Figure 9 is obtained by considering each task, and then, for each subject, by computing the percentage of positively labeled intervals (with stress) with respect to the total of intervals within the task. The figure shows the average and the standard deviation for all of the subjects, for each task. We did this for the manual and for the autonomous scenarios.

We can observe that, except during the first obstacle where the autonomous driving scenario has a slightly higher value, the manual scenario values are always considerably higher than the autonomous ones, so we can conclude that manual driving has a greater impact on SPR values and is perceived as more stressful.

**Figure 10:**
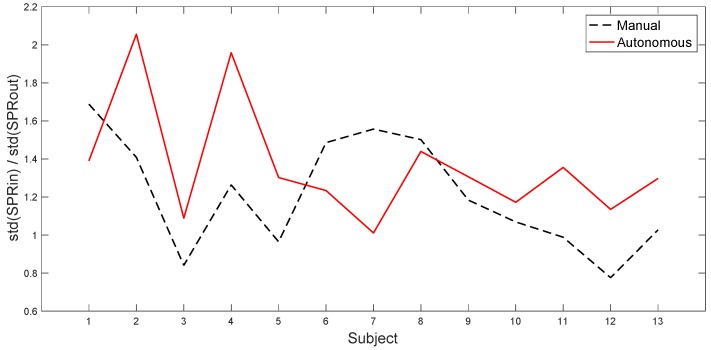
The ratio between the standard deviation of the SPR during the tasks and outside the tasks, in the manual and autonomous scenarios.

The plots in Figure 7 and Figure 9 can be explained by the fact that, as observed before, SPR peaks are less frequent in the autonomous scenario, during stress time intervals, but with a higher amplitude than in the manual scenario. Thus, the percentage of stress labels is higher in manual driving, even if the RMS values in the two scenarios are more similar. This is also confirmed in Figure 10, which shows the ratio between the standard deviation of the SPR during the tasks and outside the tasks, for each subject. The figure suggests that, for most subjects in autonomous driving, the SPR amplitude is generally lower outside of the tasks and more pronounced during the tasks. Finally, Figure 9 shows that, as a whole, the frequency of the stress intervals during the tasks is always higher in manual driving, albeit the intensity of the peaks is usually lower.

**Figure 11:**
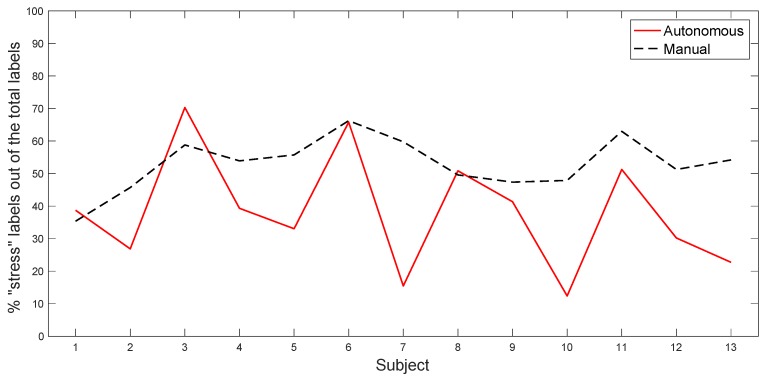
Total number of “stress” labeled intervals for each subject (in %), considering the whole test track.

Table 2 shows the total count of “stress” labeled intervals for each subject, considering the whole test track (Figure 11 is a graphical representation of the table). We can observe that the autonomous drive, in our experiment, appears to be perceived as less stressful for all of the subjects except one, and in two subjects, the values are almost equal.

Moreover, we observe that the overall path length with the tasks is 12 km, which is 30% of the total length of the track equal to 40 km. We suppose that the subjects will be stressed performing those tasks, so we expect to have at least 30% of “stress” labeled intervals among all of the subjects. This supposition seems to be confirmed by the mean values equal to 38% for the autonomous scenario and to 53% for the manual scenario.

**Figure 12:**
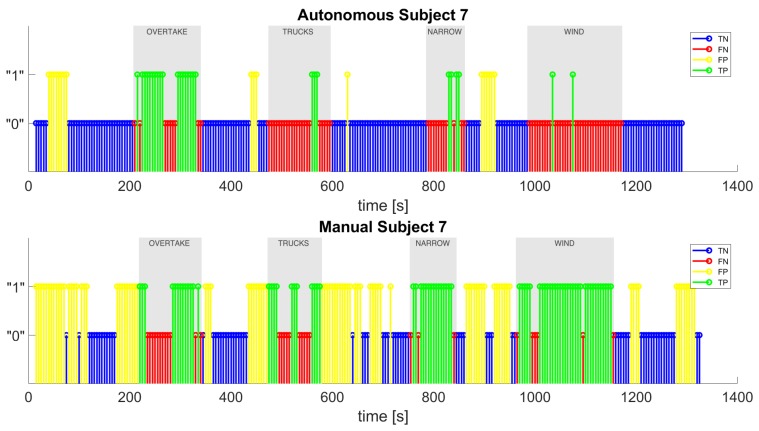
The classifier’s output for test subject 7 in autonomous and manual driving.

**Figure 13:**
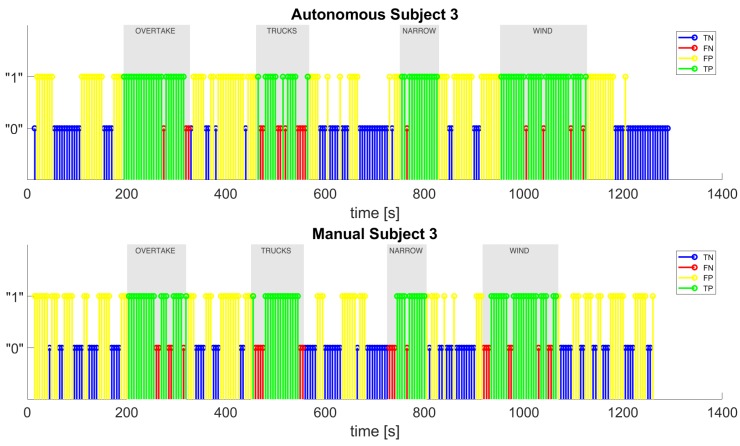
The classifier’s output for test subject 3 in autonomous and manual driving.

Finally, Figure 12 depicts an example of the classifier’s output for one of the test subjects, showing the true positives (green), false positives (yellow), true negatives (blue), and false negatives (red). The true positives are all of the intervals that the classifier labeled as “stress” correctly, because they fall into the stress episodes; the false negatives are all of the “non-stress” labeled intervals which instead should have been labeled as “stress”, because they fall into the stress episodes; the true negatives are all of the correctly labeled “non-stress” intervals, because they fall outside the stress episodes; the false positives are all of the incorrectly detected as “stress” intervals that should have been labeled as “non-stress” because they fall outside the stress episodes. As it can be noted, the total number of positive labels (the sum of true positives and false positives) is by far greater in the manual scenario than in the autonomous scenario, so we can assume that the subject is less stressed during the autonomous drive. We also show in Figure 13 the classifier’s output for the test subject in which the number of positive labels is much higher in the autonomous case than in the manual one.

### 4.3. ECG Results

**Figure 14:**
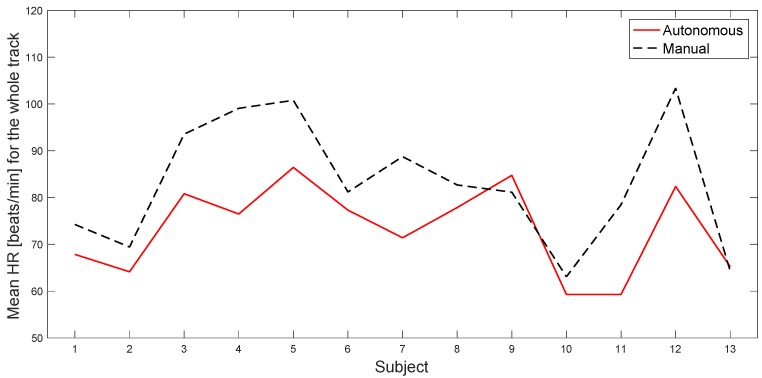
Mean value of the HR for all of the subjects during the whole track, in both autonomous and manual scenarios.

As already mentioned, we recorded the ECG signal from each subject as well. We noticed a systematic difference between the characteristics of the instantaneous Heart Rate (HR) between autonomous and manual driving tests. This is certainly due in part to the physical activity involved in manual driving, which, however, makes it difficult to directly compare the two scenarios using HR information. We nevertheless show in Table 3 the mean values of the HR during and outside the tasks, for each subject, because this also can possibly be an indication of the perceived stress level. In fact, we notice that, as stated before, the mean HR of the subjects is typically higher during the manual driving scenario for the whole track (see also Figure 14 that visually shows the mean value of the HR for all of the subjects during the whole track). In addition, we can see that the HR changes considerably between the subjects, and that in the manual scenario there is a greater variability of the HR values in all cases: for the same subject in different tasks, for the same subject on the sections of the track without tasks, and for the same subject considering the whole track. On the other hand, in the autonomous scenario, there is less variability.

## 5. Discussion and Conclusions

In this paper, we presented a system (which is under continuous development) that allowed us to evaluate the stress in drivers in a simulated manual and autonomous drive scenario by considering the SPR signal of 13 subjects. To this purpose, we developed a methodology and utilized a professional driving simulator. The subjects had to overcome four stress tasks along a drive on a highway where these events were placed. They have to go through this in two cases: by manually driving, and being idle while the autonomous drive algorithm drove for them. We apply a paired t-test to the SPR data to evaluate how statistically significant the difference is between the values coming from the autonomous and manual cases, and considering the different tasks. These findings are in agreement with those acquired using an SVM classifier, assuming that the SPR responses during autonomous and manual driving are directly comparable. Our results indicate some difference in autonomous vs. manual driving, which, however may also be influenced by the somewhat limited test data and SVM training set. In our particular setting, which needs to be confirmed by expanding the pool of subjects and increasing the scenario’s determinism, it looks like the manual driving scenario appears to be perceived as more stressful than the autonomous one. In fact, we show that SPR peaks appear less frequently in the autonomous scenario during the tasks, but with a higher peak value than in the manual scenario. Thus, the number of stress labels is typically larger in manual driving, even though the RMS values in the two cases are similar. This could be linked to the increased attention needed to manually complete a task and to the worry of making mistakes in doing that. The autonomous drive, on the other hand, might induce some degree of “fear” on the subjects. However, we also notice that for one subject this does not happen, i.e., that the number of positive labels is greater in the autonomous scenario than in the manual one, and that for two other subjects, the resulting positive labels in the manual and autonomous cases are similar. We also consider the mean value of the Heart Rate of the subjects, and we observe that the HR is generally higher in manual driving, which can be an additional indicator (within the limitations of the considered experiment) that the general perceived stress is higher during a manual drive. There is a variability in HR between the subjects, but in the manual scenario, we can observe a greater variability of the HR values in all cases: for the same subject in different tasks, for the same subject on the remaining part of the track, and for the same subject considering the track as a whole. On the other hand, in the autonomous drive scenario, the variability of the HR is much smaller. We also notice that sometimes, when peaks in the SPR signal occur, hearth rate peaks appear at the same time in the HR signal. This, however, is noticeable in manual driving but not during autonomous driving. Since HR is surely influenced by physical activity, we assumed that the two situations were not directly comparable, and we did not consider the HR signal as input to the ML classifier.

## Figures and Tables

**Table 1:** The features used for the classification algorithm.

Input Signal	Selected Features
SPR Signal	Variance
	Energy
	Mean absolute value
	Mean absolute derivative
	Max absolute derivative

**Table 2:** Total number of “Stress” intervals (in %) in manual and autonomous drive.

Manual														
Subject	1	2	3	4	5	6	7	8	9	10	11	12	13	Mean
P (%)	35.37	45.71	58.80	53.91	55.74	66.25	59.76	49.59	47.37	47.90	62.98	51.26	54.20	52.99
**Autonomous**														
Subject	1	2	3	4	5	6	7	8	9	10	11	12	13	Mean
P (%)	38.72	26.86	70.29	39.33	33.05	65.81	15.48	50.85	41.35	12.39	51.27	30.21	22.73	38.34

**Table 3:** Mean HR in manual and autonomous drive.

Manual														
Subject	1	2	3	4	5	6	7	8	9	10	11	12	13	Mean (bpm)
task 1	75.96	73.64	100.17	109.08	108.23	83.11	97.41	85.98	79.80	63.29	79.60	99.34	64.08	86.13
task 2	76.11	74.08	99.04	110.09	108.78	83.45	97.19	128	85.20	79.97	79.69	100.58	64.22	86.28
task 4	76.63	74.43	100.04	109.84	109.13	83.39	97.58	85.98	79.98	63.06	80.16	100.09	64.10	86.49
task 4	76.12	74.02	99.21	109.68	108.62	83.35	97.23	85.35	79.98	63.21	79.69	100.41	64.05	86.22
no tasks	74.10	68.98	92.80	97.83	99.92	80.97	87.72	82.38	81.33	63.07	78.40	103.83	64.29	82.74
whole track	74.25	69.44	93.55	99.07	100.78	81.20	88.75	82.70	81.15	63.09	78.48	103.38	64.27	83.09
**Autonomous**														
Subject	1	2	3	4	5	6	7	8	9	10	11	12	13	Mean (bpm)
task 1	66.64	65.89	81.10	77.92	86.81	78.29	73.91	74.99	81.55	60.66	82.03	81.58	64.26	75.05
task 2	66.47	65.98	81.29	77.75	86.66	78.50	73.84	74.96	81.79	60.80	81.96	81.89	64.32	75.09
task 3	66.49	65.98	81.29	77.78	86.68	78.48	73.85	74.96	81.76	60.79	81.98	81.87	64.34	75.10
task 4	66.64	65.89	81.08	77.90	86.81	78.29	73.93	74.99	81.53	60.64	82.02	81.56	64.25	75.04
no tasks	68.02	63.96	80.78	76.33	86.40	77.19	71.11	78.24	85.12	59.11	85.95	82.44	65.32	75.38
whole track	67.86	64.14	80.82	76.49	86.44	77.31	71.41	77.87	84.75	59.29	85.52	82.37	65.22	75.35

## Abbreviations

The following abbreviations are used in this manuscript:

SPR	Skin Potential Response
EDA	Electrodermal Activity
ECG	Electrocardiogram
MA	Motion Artifact
ML	Machine Learning
SVM	Support Vector Machine
